# Interrogating Encapsulated Protein Structure within Metal–Organic Frameworks at Elevated Temperature

**DOI:** 10.1021/jacs.2c13525

**Published:** 2023-03-24

**Authors:** Rohan Murty, Mrinal K. Bera, Ian M. Walton, Christina Whetzel, Mark R. Prausnitz, Krista S. Walton

**Affiliations:** † School of Chemical and Biomolecular Engineering, Georgia Institute of Technology, Atlanta, Georgia 30332, United States; ‡ NSF’s ChemMatCARS, Pritzker School of Molecular Engineering, The University of Chicago, Chicago, Illinois 60637, United States

## Abstract

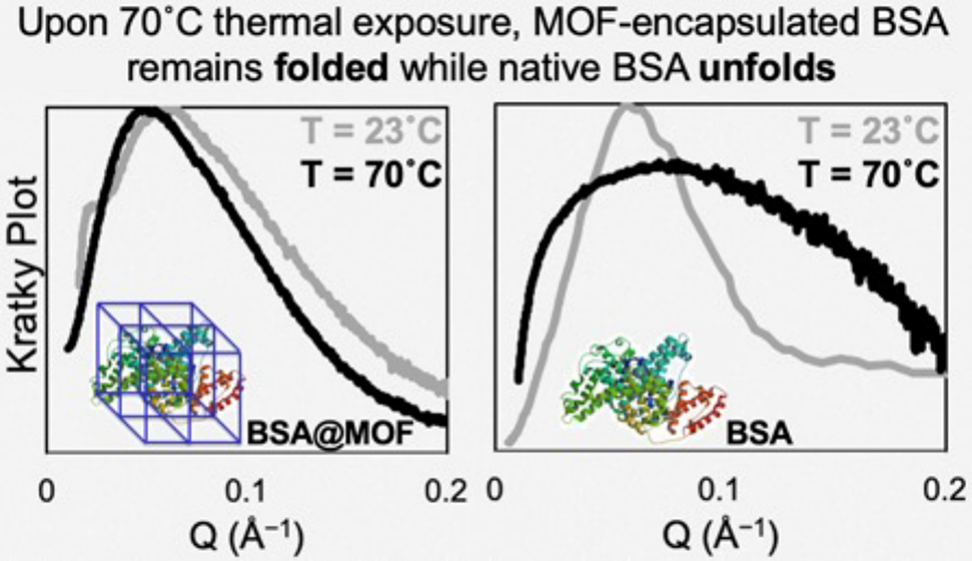

Encapsulating biomacromolecules within metal–organic frameworks (MOFs) can confer thermostability to entrapped guests. It has been hypothesized that the confinement of guest molecules within a rigid MOF scaffold results in heightened stability of the guests, but no direct evidence of this mechanism has been shown. Here, we present a novel analytical method using small-angle X-ray scattering (SAXS) to solve the structure of bovine serum albumin (BSA) while encapsulated within two zeolitic imidazolate frameworks (ZIF-67 and ZIF-8). Our approach comprises subtracting the scaled SAXS spectrum of the ZIF from that of the biocomposite BSA@ZIF to determine the radius of gyration of encapsulated BSA through Guinier, Kratky, and pair distance distribution function analyses. While native BSA exposed to 70 °C became denatured, in situ SAXS analysis showed that encapsulated BSA retained its size and folded state at 70 °C when encapsulated within a ZIF scaffold, suggesting that entrapment within MOF cavities inhibited protein unfolding and thus denaturation. This method of SAXS analysis not only provides insight into biomolecular stabilization in MOFs but may also offer a new approach to study the structure of other conformationally labile molecules in rigid matrices.

## Introduction

Engineering protein stability is an important and persistent challenge in living systems.^1^ From engineering enzyme robustness to reducing the rate of cold-chain failures during vaccine transport, many subfields would benefit from a simple, abstractable platform that renders proteins more stable.^2,3^ Metal–organic frameworks (MOFs)—a class of highly porous materials used for separations, catalysis, and drug delivery applications—may offer one such solution.^4−7^ In recent years, MOFs have emerged as a possible solution for enhancing biomacromolecule stability through an encapsulation process known as biomimetic mineralization. With this approach, prior studies have demonstrated the retention of protein and virus stability after exposure to normally denaturing temperatures as high as 80 °C.^8,9^

Heightened stability of a guest molecule (e.g., protein) due to confinement within a porous host structure has also been observed in other biocomposites such as mesoporous silicas.^10,11^ In contrast to the silica biocomposites, due to the size of bovine serum albumin (BSA) (∼6 nm diameter) and the zeolitic imidazolate framework (ZIF) pore size (∼1 nm diameter), guest encapsulation occurs within larger cavities formed by MOF growth around the guest, not confinement within a single MOF pore.^8^ For both MOFs and silicas, quantifying the guest structure—while still encapsulated—would be advantageous, especially during in situ heating, as this may impart clarity on the (still unconfirmed) mechanism of thermal stability. Because biocomposite guest stability is conventionally measured upon guest release from the porous framework (e.g., through exfoliation by ionic buffers, acids, and/or chelating agents), there are relatively few prior studies examining the encapsulated guest structure.^12,13^

Some groups have used site-directed spin labeling electron paramagnetic resonance (SDSL-EPR) to resolve the structure of enzymes embedded in both MOFs and covalent organic frameworks.^14,15^ These studies, bolstered by protein simulation and modeling, mapped the orientation and degrees of freedom of embedded guest enzymes at ambient temperature. Solid-state nuclear magnetic resonance (NMR) has also been used to examine surface interactions between guests and host frameworks in MOF biocomposites, revealing changes in the coordination of metal centers upon guest encapsulation.^16^ However, neither SDSL-EPR nor NMR studies have examined the guest structure during thermal exposure, an important factor for biopreservation applications. One study on a MOF-encapsulated enzyme showed that guest stability was retained after biocomposite heating to 70 °C.^17^ This work, however, left the thermally exposed guest’s size and structure unresolved, as it used the enzymatic activity of the guest as a proxy for stability. Because most studies thus far have focused on specific host–guest interactions and have not directly resolved guest morphology during in situ heating, there exists a fundamental gap in the MOF biocomposite literature. The approach presented in this work addressed this gap by providing a novel method to directly measure guest properties, notably during in situ thermal exposure.

In this study, we employed small-angle X-ray scattering (SAXS) to resolve the morphology of still-encapsulated guests. SAXS is an established technique for analyzing nanoparticles and biological macromolecules in solution.^18,19^ When coupled with synchrotron-source radiation, SAXS becomes a robust method for resolving the size, structure, and oligomeric state of proteins.^20−23^ Furthermore, this method can be used to observe a protein’s structural changes in real-time, allowing the study of protein stability even during in situ heating.^24,25^ SAXS data analysis techniques like the Guinier and Kratky plots are generally employed to reveal the protein’s radius of gyration and degree of folding, respectively.^26,27^ Furthermore, the most used SAXS analysis tool is the pair distance distribution function (PDDF), a frequency plot of interatomic distances mathematically derived from the raw intensity plot.^28^ Interpretation of the PDDF can allow the assessment of a protein’s geometry and tertiary structure.^29^

Guided by prior studies of unencapsulated proteins, we demonstrate here that synchrotron-source SAXS measurements on protein–MOF biocomposites may allow the structural analysis of still-encapsulated guest molecules. We achieved this by scaling the pure MOF SAXS spectra by a scalar value and subtracting it from the raw intensity plot of the MOF biocomposite. In this approach, the scaling subtraction factor may be determined analytically using physical characteristics of the biocomposite system or empirically by inspection of the raw SAXS spectra. After the background subtraction, standard SAXS analysis techniques (i.e., Guinier, Kratky, and PDDF) were applied to study the structure of the encapsulated protein. Other groups have used SAXS to analyze MOF biocomposites, but these reports typically study the growth and/or structure of the reticular host material; our work specifically focuses on the size, structure, and folding of the guest molecules.^8,30,31^

The biocomposites reported here were composed of BSA encapsulated within two ZIFs named ZIF-67 and ZIF-8. These frameworks are in a subfamily of MOFs composed of the ligand 2-methyl imidazole and either cobalt or zinc as the metal center, forming the isostructural ZIF-67 or ZIF-8, respectively.^32,33^ BSA was selected as the guest species due to its ubiquity and well-characterized denaturation behavior.^34^ ZIF-67/ZIF-8 were selected as the hosts due to their good stability in water and accessibility by room-temperature, aqueous synthesis—both with and without the guest molecule present.^35,36^ This is notable as many MOF syntheses involve temperatures and/or solvents that could damage a proteinaceous guest.^37^

By performing SAXS measurements on the synthesized biocomposites (BSA@ZIF-67 and BSA@ZIF-8) and then comparing these results to physical mixtures of pure MOF species and BSA, we determined the ideal conditions for our spectral subtraction approach. We then conducted SAXS measurements during in situ heating experiments past the denaturation temperature of BSA using a specialized sample holder, which demonstrated BSA stability resulting from MOF encapsulation.

## Methods

### SAXS with and without In Situ Heating

Solid samples were loaded into 1.5 mm O.D. quartz capillary tubes (Charles Supper Company, Westborough, MA, USA) and then suspended by pipetting 100 mM HEPES buffer into the capillaries and agitating to improve homogeneity within the scattering volume. The mildly basic pH of HEPES (7.4) and its negligible effect on ZIF stability justified its choice as a buffer solution.^38^ BSA and buffer solutions were pipetted directly into capillaries.

SAXS measurements were done by loading the capillary tubes on a capillary tube holder either with or without temperature control. All measurements were done with 20 keV X-rays with wavelength (λ) 0.62 Å at the 15-ID-D station of NSF’s ChemMatCARS (Sector 15), Advanced Photon Source. The scattered X-rays were measured with Pilatus 3X 300 K area detector with a 1 mm silicon chip and a sample-to-detector distance of 3.67 m. The images from the area detector were reduced to one-dimensional *q* vs *I* curves by azimuthal integration, where *q* = (4π/λ) × sin(θ/2) is the reciprocal lattice vector and θ is the scattering angle. The ambient temperature SAXS measurements were done at ∼23 °C, and the high-temperature measurements were done at 70 °C.

### Background Subtraction

In conventional SAXS experiments, background subtraction of the solvent from the protein solution is necessary for meaningful data analysis.^39^ While this approach works well in two-component systems where both the volume fractions are known, we employed an empirical approach in our scaled spectral subtraction because the biocomposite system has three components: the protein, the MOF structure, and the solvent with unknown fractions in the scattering volume. Further analytical justification of the empirical background subtraction is provided in the Supporting Information. Assuming a constant background from the solvent in the *Q*-range of analysis, we can scale the scattering from the MOF with the solvent with a scaling factor, α, and subtract that from the biocomposite in water to calculate the encapsulated protein spectra. Accurate subtraction of pure MOF spectra from the biocomposite is highly dependent on carefully selecting the scaling subtraction factor, α. For the BSA in buffer solution, a two-component system, the buffer solution background scattering was subtracted by established methods.^40^ For the biocomposite three-component systems, we scaled the pure MOF (in buffer solution) spectra by α values ranging between 1 and 20. The initial α value was informed by the protein mass ratio within that specific biocomposite (20–25%) but almost always had to be empirically adjusted due to variations in the density of the suspension, heterogeneity within the scattering volume, variable thickness of the capillary tubes, etc. The scaled pure MOF spectra (in buffer solution) were then subtracted from the biocomposite to yield the encapsulated protein spectra, and the α values were adjusted to minimize the sum-squared error between the native and the encapsulated protein spectra (as shown in Figure 2). Once the encapsulated protein spectral intensity was brought to the scale of the native raw protein spectra, we performed PDDF, Guinier, and Kratky analyses for further structural determination. By comparing the PDDF, Guinier, and Kratky analyses of the encapsulated protein spectra with that of the native protein, we inferred whether the encapsulated protein retained the native structure within the MOF structure.

### Pair Distribution Function and Guinier Analysis

After performing background and/or MOF subtraction, the datasets were then imported into SasView (www.sasview.org), an open-source analysis program used to generate pair distribution functions and Guinier fits. When inverting SAXS spectra into PDDFs, a *Q*-range of 0.04–0.25 Å^–1^ was considered with a maximum interatomic distance of 100 Å. For fitting, regularization constants ranging from 1 × 10^15^ to 1 × 10^19^ were used.

## Results

### Confirming BSA Encapsulation within ZIFs

**Figure 1:**
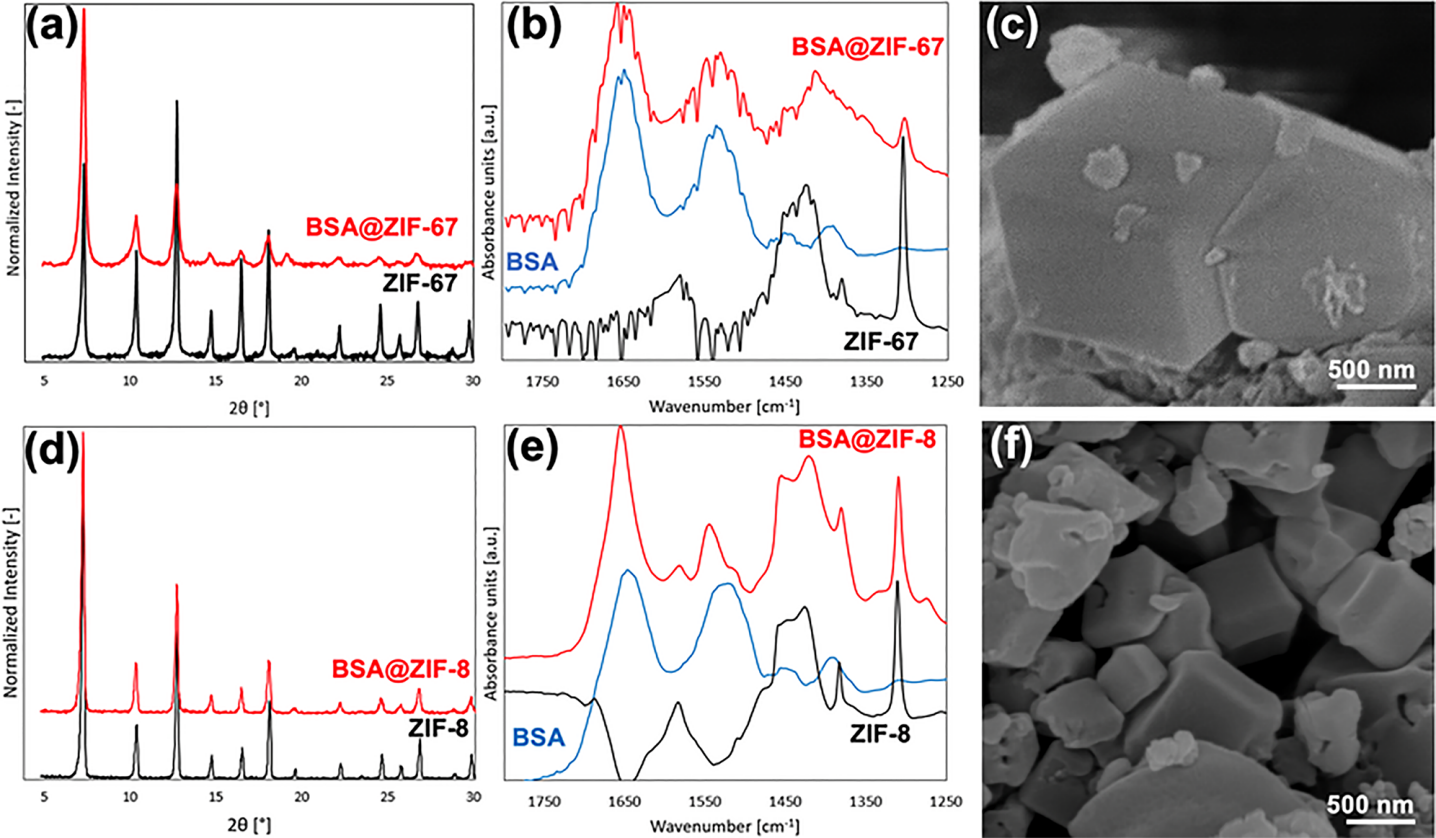
Characterization of BSA@ZIF-67/ZIF-8 biocomposites. Representative XRD spectra of (a) BSA@ZIF-67 and (d) BSA@ZIF-8 showing the biocomposite (red) and MOF (black). Representative FTIR spectra of (b) BSA@ZIF-67 and (e) BSA@ZIF-8, showing the biocomposite (red), lyophilized native BSA protein (blue), and MOF (black). Representative SEM images of (c) BSA@ZIF-67 and (f) BSA@ZIF-8.

Before SAXS analyses, the synthesized biocomposites were characterized with X-ray diffraction (XRD), Fourier transform infrared spectroscopy (FTIR), and scanning electron microscopy (SEM) to confirm that BSA had been successfully embedded into the ZIF framework and that this structural incorporation did not disrupt the long-range order or general morphology of the host material (Figure 1).

Diffractograms for both the biocomposites (BSA@ZIF-8 and BSA@ZIF-67) and pure MOFs (ZIF-8 and ZIF-67) were similar to each other and showed strong agreement with the simulated pattern (Figure 1a,d), as well as those previously reported in the literature for ZIF-8 and ZIF-67.^41,42^ The biocomposite patterns were nearly identical to those of the pure ZIF, except for a slight decrease in the apparent signal-to-noise ratio. This behavior has been observed in other biocomposites formed from proteins and MOFs^8,43^ and is likely caused by heterogeneous (i.e., anisotropic) incorporation of BSA within the host framework, resulting in mild disruption of the constructive scattering. Nonetheless, these crystallography results provide compelling evidence that the long-range order of the host scaffold was not significantly compromised by protein encapsulation.

FTIR spectrograms of pure ZIFs contain a characteristic plateau from 1475 to 1400 cm^–1^ wavenumbers caused by the methyl bending mode from the HMe-Im ligands^44^ (Figure 1b,e). For the lyophilized BSA spectrogram, there are two proteinaceous peaks centered at 1650 and 1550 cm^–1^; these result from the protein’s C=O and N–O stretching modes, respectively. Notably, the pure ZIF-8 and ZIF-67 spectra do not contain either of these proteinaceous peaks as the framework lacks carbonyl and nitro groups. Finally, the spectrograms of the biocomposites BSA@ZIF-8 and BSA@ZIF-67 contain the carbonyl/nitro peaks of the protein as well as the characteristic plateau of the pure MOF. Because the two proteinaceous peaks experience a mild upward shift in wavenumber (∼25 cm^–1^) when seen in the biocomposite, there is likely a weak (non-covalent) interaction between the protein and the MOF, suggesting that surface-bound BSA is not responsible for those peaks. Additionally, because the crystallites were washed vigorously with both water and ethanol before analysis, we expect that the proteinaceous peaks in the biocomposite are a result of protein encapsulation in the MOF. Further evidence for the success of the surface wash is afforded by an enzyme-linked immunosorbent assay (ELISA) specific to BSA (Figure S1). Intact biocomposite particles showed a low BSA concentration as embedded BSA could not interact with the plate wells. Meanwhile, BSA@ZIF-8 exfoliated by EDTA showed a dramatic increase in the BSA concentration, again demonstrating the release of the encapsulated BSA from the MOF.

SEM imaging shows MOF biocomposite crystals, which exhibit a characteristic rhombic dodecahedron geometry with an edge length of ∼1 μm; this is expected of ZIF-8 and ZIF-67 crystallites, as reported extensively in the literature.^45^ The biocomposite crystallites have smooth surfaces showing no evidence of surface-bound proteins, which further suggests that the wash protocol was successful, confirming BSA encapsulation within the framework (Figure 1c,f). Further evidence of encapsulation can be gathered by comparing the BSA@ZIF crystallite and pure ZIF crystallite size (Figure S2). Crystallites of pure ZIF-67 (Figure S2a) and ZIF-8 (Figure S2b) are much smaller (100–200 nm) than the synthesized biocomposites, suggesting that guest proteins have a pronounced effect on crystal growth.

### Subtraction Approach Reveals SAXS Spectra of Encapsulated BSA within Biocomposites

**Figure 2:**
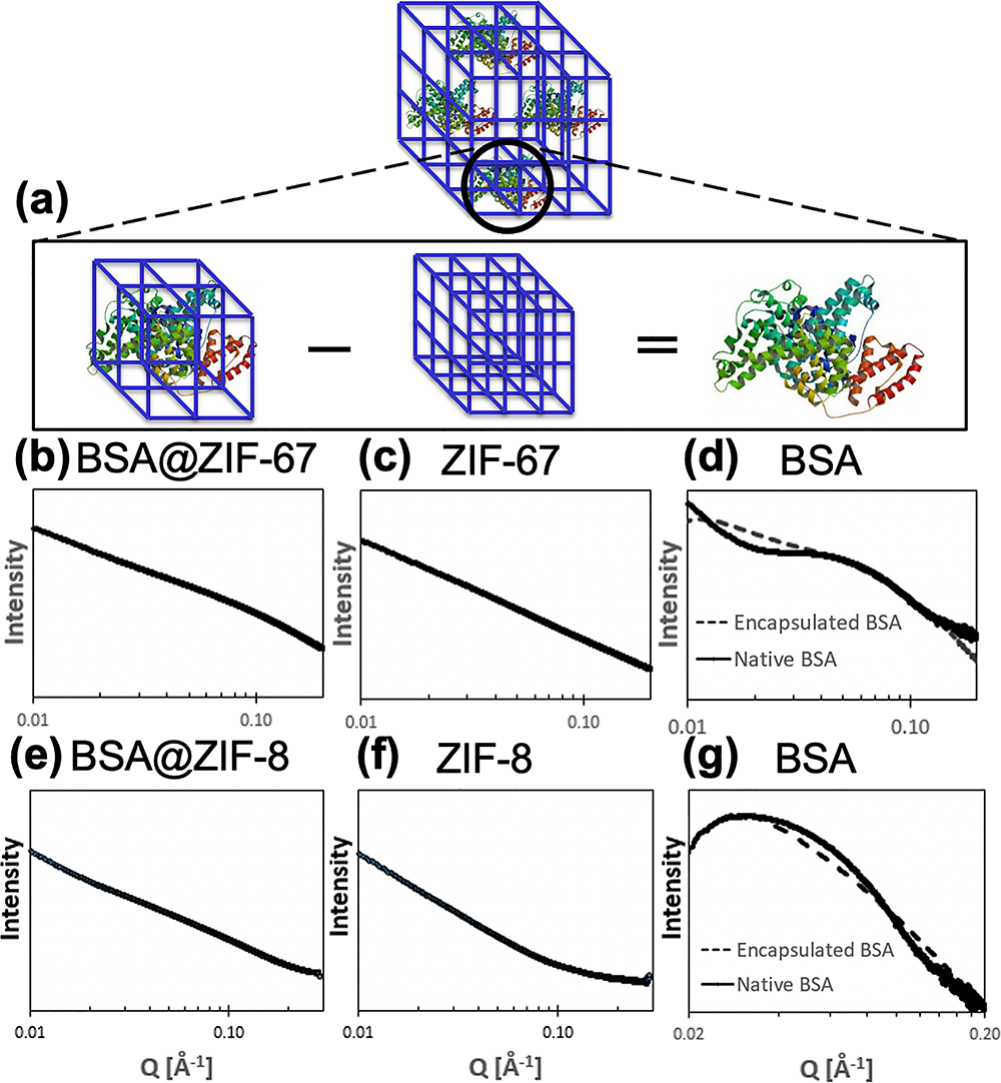
Schematic of (a) scaled spectral subtraction to reveal encapsulated BSA in ZIF biocomposites. Log–log scale SAXS spectra are shown for (b) BSA@ZIF-67, (c) ZIF-67, (d) native or encapsulated BSA in ZIF-67, (e) BSA@ZIF-8, (f) ZIF-8, and (g) native or encapsulated BSA in ZIF-8. Spectra for encapsulated BSA (d, g) were generated by subtracting the ZIF (c, f) spectra from the BSA@ZIF (b, e) spectra and compared to native BSA in buffer solution. Before subtracting MOF spectra from biocomposite spectra, solvent background subtraction was completed for all spectra. ZIF-8 and ZIF-67 spectra were subtracted from their corresponding biocomposite spectra using scaling subtraction factors of 5 and 20, respectively. The *Q*-range is shown from 0.01 to 0.20 Å^–1^. BSA@ZIF-8 and BSA@ZIF-67 were prepared at BSA/MOF ratios of 3:1 in HEPES buffer. The native BSA was prepared at a concentration of 4 mg/mL in HEPES buffer.

The SAXS spectra for the biocomposites (BSA@ZIF-8 and BSA@ZIF-67) and the pure MOFs (ZIF-8 and ZIF-67) are nearly indiscernible by visual inspection; this is owed largely to the MOF dominating the scattering in the *Q*-range shown (Figure 2b,c,e,f). There were, nonetheless, differences between the biocomposite and pure MOF spectra, which were revealed by subtracting the pure MOF spectrum from the biocomposite spectrum (Figure 2d,g). The calculated “encapsulated BSA” spectra are in good agreement with the “native BSA” spectra, suggesting that the empirical scaled spectral subtraction approach can provide reasonable results.

The biggest variation in the encapsulated and native BSA SAXS spectra is seen in the *Q*-range of 0.01–0.03 Å^–1^ and is likely explained by BSA aggregation in solution that is inhibited when confined in the MOF. Indeed, when comparing native and encapsulated BSA (Figure S3), it is apparent that native BSA shows evidence of aggregates in solution indicated by the presence of oligomers (i.e., of larger size), whereas encapsulated BSA is almost completely monomeric (i.e., with one protein occupying one cavity each). Both interpretations are consistent with previous reports.^8,46^

Successful subtraction to calculate encapsulated protein spectra in both ZIF-67 and ZIF-8 suggests that this approach may have broad applicability. We note, however, that both MOF species reported here have isostructural sodalite topologies, indicating that further study of biocomposites with varying guests, hosts, and topologies is necessary to more fully assess breadth of applicability of our approach.

**Figure 3:**
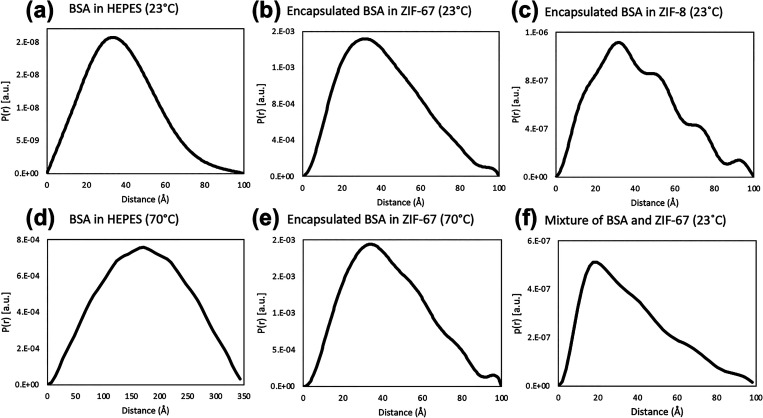
Representative PDDFs for BSA, BSA@ZIF-67/ZIF-8, and a physical mixture of BSA and ZIF-67 prepared at a BSA/MOF ratio of 1:4 (20% BSA). PDDFs shown for (a) 4 mg/mL native BSA in HEPES buffer at 23 °C, (b) calculated spectra of BSA encapsulated in ZIF-67 at 23 °C, (c) calculated spectra of BSA encapsulated in ZIF-8 at 23 °C, (d) 4 mg/mL native BSA in HEPES buffer at 70 °C, (e) calculated spectra of BSA encapsulated in ZIF-67 at 70 °C, and (f) calculated spectra of BSA from a physical mixture of lyophilized BSA and ZIF-67. *R*_g_ values calculated from PDDFs were (a) 29.53 ± 0.18 Å for native BSA at 23 °C, (b) 32.21 ± 0.06 Å for ZIF-67 encapsulated BSA at 23 °C, (c) 32.57 ± 0.46 Å for ZIF-8 encapsulated BSA at 23 °C, (d) 133.0 Å for native BSA at 70 °C, (e) 33.34 ± 0.04 Å for ZIF-67 encapsulated BSA at 70 °C, and (f) 29.9 Å for the physical mixture at 23 °C. Corresponding SAXS spectra are shown in Figure 2.

We next used these subtracted spectra to generate PDDFs, which provide a more quantitative representation of the encapsulated protein’s size and geometry compared to SAXS spectra and allow the calculation of the protein molecule’s radius of gyration (*R*_g_) (Figure 3).

The PDDFs for encapsulated and native BSA are in good agreement with each other and indicate that BSA assumed the expected globular (spherical) conformation, as indicated by the bell-shaped curve shown in the PDDF (Figure 3a,b).^47^ The *R*_g_ values calculated from the PDDFs were all approximately 30 Å and were consistent with previous literature studies on free-standing BSA.^46,48^ This was true for the calculated spectra of BSA encapsulated in ZIF-67 (Figure 3b) and ZIF-8 (Figure 3c). We also performed Guinier fits on BSA encapsulated in ZIF-67 and native BSA, which further improved our confidence in the successful subtraction (Figure S4). The *R*_g_ values calculated with PDDF and Guinier analyses were similar but varied slightly possibly due to minute differences in the SAXS spectra at the low-*Q* region, which is also reflected by slight differences in the PDDFs at larger distances. These minor inconsistencies could be due to the interaction of the protein surface with the interior cavity walls of MOFs. Altogether, the observation of very similar structural parameters of proteins within ZIF-8 and ZIF-67 provides confidence that the scaled spectral subtraction method can calculate the encapsulated protein spectra and *R*_g_ values, allowing in situ SAXS-based analysis of embedded guests for the first time.

### Subtraction Approach Applied to a Physical Mixture of Proteins and MOFs

The scaled spectral subtraction approach should similarly work to isolate the SAXS signal from lyophilized BSA in a physical mixture with pure MOF suspended together in buffer (Figure 3f). This expectation appeared generally to be correct as the calculated PDDF from a mixture of BSA and ZIF-67 produced a characteristic shape expected for a protein, although with a somewhat flattened shape consistent with a cylindrical protein conformation rather than the more-rounded shape associated with globular proteins.^47^ The predicted *R*_g_ value was 29.9 Å, in good agreement with the expected value.^46^ Nonetheless, this cannot be considered a successful subtraction since both the *R*_g_ and PDDF must be consistent with the native protein. The difference could be explained by protein aggregation in the solution or an insufficiently high protein mass ratio in the mixture.

It is also worth noting that this experiment used biocomposites composed of 20–25% BSA, which provided sufficient signal from the protein to generate a calculated spectrum in agreement with pure BSA spectra. When we conducted a similar experiment using a mixture of BSA and ZIF-67 with a BSA/MOF ratio of just 1:9 (10% BSA), the pure BSA PDDF was poorly reproduced, and the data had large error bars (Figure S5).

### Subtraction Approach during In Situ Heating of Biocomposites

The development of the scaled spectral subtraction approach was motivated by the need to assess the size and conformation of the encapsulated protein as a measure of protein stability. Therefore, we subjected BSA to a thermal stress of 70 °C for 3 h both as native BSA and BSA@ZIF-67 (Figure 3d,e). Comparison of BSA@ZIF-67 at 70 and 23 °C (Figure 3e,b) showed no meaningful change in the PDDF of the encapsulated protein, indicating a stable protein conformation. *R*_g_ of BSA@ZIF-67 at 70 °C was 33.34 ± 0.04 Å, which varied only slightly from *R*_g_ of the biocomposite at 23 °C (32.21 ± 0.06 Å). These findings suggest that tight confinement of the protein guest within MOF cavities prevented its unfolding and is at least partially responsible for increased thermostability caused by the encapsulation.

In contrast, a comparison of native BSA at these two temperatures (Figure 3d,a) showed a marked change in the PDDFs, indicating dramatic changes to the protein conformation. This is not surprising as the denaturation temperature of BSA in solution is known to be 65 °C.^34^ Extended exposure to denaturing temperatures is expected to lead to loss of globular conformation and protein aggregation, as evidenced both by PDDF broadening for the denatured BSA as well as visual inspection of the capillary tubes during in situ heating (data not shown). Additionally, the *R*_g_ value at 70 °C increased to 133.0 Å, which was much greater than the value at 23 °C (29.53 ± 0.18 Å). This behavior is consistent with protein unfolding and aggregation as well as previous SAXS studies on the denaturation of BSA at 70 °C.^46^

**Figure 4:**
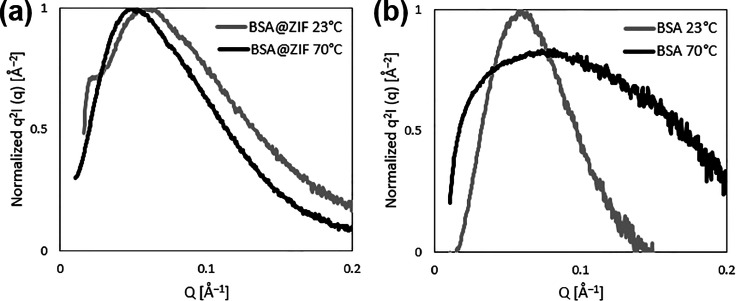
Representative Kratky plots of encapsulated and native BSA at 23 and 70 °C. Kratky plots from the calculated spectra of (a) BSA@ZIF-67 as a dry powder and (b) native BSA in HEPES buffer at a concentration of 4 mg/mL heated to 70 °C for 3 h. Corresponding PDDFs at 23 °C are shown in Figure 3a,b, and those at 70 °C are shown in Figure 3d,e.

To further assess the thermostability of BSA@ZIF-67, we conducted Kratky analysis of the calculated SAXS spectra, which provides a qualitative way to assess the nature of protein folding by plotting the SAXS intensity *I*(*q*) multiplied by *Q*^2^ versus *Q*. In this approach depicted in Figure 4, Kratky plots of unknown samples are referenced against known Kratky plot shapes to assess the folded nature of proteins.^49^

The Kratky plots for BSA@ZIF-67 at 23 and 70 °C show again that at both ambient and denaturing temperatures, the encapsulated BSA protein remained folded within the MOF cavities as both curves exhibit the characteristic “folded” conformation characteristic of Kratky plots^46^ (Figure 4a). The Kratky plots also confirm that exposing BSA to elevated temperatures led to extensive unfolding, manifesting as loss of the characteristic peak associated with folded proteins (Figure 4b). Differences in the Kratky plot shapes for encapsulated and native BSA at room temperature could again be due to the interaction of the protein with the cavity walls of MOFs, as also observed from the *R*_g_ values from PDDF analyses. Thus, the combined insights from PDDF and Kratky analyses indicate that tight confinement of proteins within MOF cavities is associated with heightened thermostability that avoids protein conformational changes leading to denaturation.

## Discussion

This study made advances in two areas: the introduction of a new method to analyze encapsulated proteins in situ by SAXS and the understanding of the protein stabilization mechanism by MOF encapsulation. First, we developed a method to calculate the SAXS spectra of a proteinaceous guest encapsulated within a MOF scaffold based on the expectation that biocomposite scattering is an additive combination of the scatterings from the protein, MOF, and solvent. Using this approach, we were able to generate the calculated SAXS spectra as well as the corresponding PDDFs, Guinier fits, and Kratky plots for BSA@ZIF-8 and BSA@ZIF-67 that showed good agreement with native BSA protein data.

We additionally showed that the subtraction approach yielded similar results for physical mixtures of BSA and ZIF-67, indicating that possible interactions between the encapsulated protein guest and the MOF host did not alter the SAXS spectra in meaningful ways. The fact that our approach worked for a mixture of BSA and ZIF suggests that successful subtraction cannot be taken as evidence of encapsulation; for this, material characterization techniques like FTIR and XRD should be employed. Consequently, we would expect the BSA present in the mixture of BSA and ZIF-67 to denature when exposed to 70 °C for 3 h.

Accurate subtraction of the MOF spectra to calculate encapsulated protein spectra was highly dependent on carefully selecting the scaling subtraction factor, α. In our analysis, we determined α through graphical and visual methods. This method to determine α provided a rough optimization that could be improved upon by more rigorous statistical methods and possibly by first-principle calculations that account for variation in experimental conditions. Although we adopted a trial-and-error approach for determining α in this work, we also propose a mathematical basis for this scaling subtraction factor in the Supporting Information.

This work represents an important contribution to the field of reticular biocomposites. For the first time, the scaled spectral subtraction method enabled us to assess the size, shape, and folding of encapsulated proteins during thermal exposure while they are still embedded within the MOF matrix. The ability to employ this approach during in situ heating provided insight into the mechanism of heightened guest thermostability afforded by MOF encapsulation, which has been hypothesized, but not shown, to be associated with physical immobilization of the protein to prevent changes to the protein structure.^8,9^ By directly observing that encapsulated guests retained their size (evidenced by *R*_g_ consistent with native BSA) and conformation (shown by globular PDDF shapes) during exposure to elevated temperatures, our data indicate that tight confinement of guest molecules within MOF cavities is associated with, and may be responsible for, retaining protein structure and stability.

Because the subtraction approach was demonstrated successfully for BSA encapsulated in two isostructural MOF species, our findings may have implications for a variety of fields employing biocomposites made from various proteins encapsulated in MOFs or other host structures. Our data indicate that if the biocomposite scattering can be approximated as an additive combination of the pure MOF and protein spectra (i.e., no significant changes in the SAXS spectra due to protein–MOF structural correlations), the approach described here may be more broadly applicable to similar host–guest systems; future studies should address its application to other guests, hosts, environmental conditions, and topologies, such as ZIF-8 diamondoid.^50^ Greater insight into the stabilization mechanism associated with protein encapsulation may improve the rational design of MOF biocomposites used in vaccine storage, enzymatic reactions, and other applications, benefitting global populations, researchers, and industrial processes.

## Conclusions

This study introduced a new method that enabled the calculation of SAXS spectra associated with proteins encapsulated in MOF host matrices measured in situ. The method was based on empirical scaled subtraction of the spectrum of a pure MOF from the spectrum of the biocomposite to yield the spectral contribution of the protein. The approach was shown to be valid for BSA encapsulated in ZIF-8 and ZIF-67. BSA@ZIF-67 biocomposites and physical mixtures of BSA and ZIF-67 produced similar SAXS spectra, suggesting that successful subtraction is not evidence of encapsulation. Finally, the scaled spectral subtraction approach was used to show that the BSA conformation when encapsulated in ZIF-67 was unchanged during heating to 70 °C for 3 h, while pure BSA without MOF encapsulation was fully denatured and unfolded. This finding suggests that protein thermostability by MOF encapsulation may be due to the physical entrapment of the protein that prevents conformational change. The results of this study could enable future in situ SAXS analyses performed on proteins or other compounds encapsulated in MOFs (as well as other host matrices) to advance fundamental research and translation into applications involving protein encapsulation and stabilization.

## Supporting Information Available

MOF biocomposite synthesis procedures; experimental details (XRD, FTIR, SEM, and ELISA); mathematical basis for the scaling subtraction factor; ELISA for BSA@ZIF-8; SEM for pure ZIFs; Guinier approximations for BSA and BSA@ZIF-67; and PDDF for the BSA + ZIF-67 mixture (PDF)

## Abbreviations

BSA@ZIF-67: BSA encapsulated in ZIF-67
BSA@ZIF-8: BSA encapsulated in ZIF-8
PDDF: pair distance distribution function
*R*_g_: radius of gyration
SAXS: small-angle X-ray scattering
ZIF: zeolitic imidazolate framework
