# Supplementary material for: Interrogating Encapsulated Protein Structure within Metal–Organic Frameworks at Elevated Temperature
Source: J Am Chem Soc. 2023 Mar 24;145(13):7323–30. doi: 10.1021/jacs.2c13525 (PMC10080685; doi:10.1021/jacs.2c13525)
Supplement: Supplementary file 1 — ja2c13525_si_001.pdf [file ja2c13525_si_001.pdf]

SUPPORTING INFORMATION

Interrogating Encapsulated Protein Structure Within Metal-Organic Frameworks at Elevated Temperature

Rohan Murty[1], Mrinal K. Bera[2], Ian M. Walton[1], Christina Whetzel[1], Mark R. Prausnitz[1,*], Krista S. Walton[1,*]

[1] School of Chemical and Biomolecular Engineering, Georgia Institute of Technology, Atlanta, GA 30332. United States.

[2] NSF's ChemMatCARS, Pritzker School of Molecular Engineering, The University of Chicago, Chicago, IL 60637, United States

*Corresponding authors: krista.walton@chbe.gatech.edu and prausnitz@gatech.edu

Table of contents:

1. Materials and Methods (page 2)

2. Mathematical justification for subtraction approach (page 4)

3. Supplementary Figures 1 to 5 (page 5)

1. Materials and Methods
1.1 Materials
Bovine Serum Albumin (≥ 96%, Sigma-Aldrich, St. Louis, MO, USA), Triethylamine (TEA, 98%, Sigma-Aldrich), Ethanol (90%, Sigma-Aldrich), HEPES (4-(2-hydroxyethyl)-1-piperazineethanesulfonic acid) (99.5%, Sigma-Aldrich), Zinc (II) Acetate Dihydrate (≥ 98%, Sigma-Aldrich), Zinc (II) Nitrate Hexahydrate (98%, Sigma-Aldrich), Cobalt (II) Nitrate Hexahydrate (≥ 97.7%, Alfa Aesar, Ward Hill, MA, USA), 2-methyl imidazole (≥ 98%, TCI, Tokyo, Japan). Deionized (DI) water was supplied by an in-house system (Thermo Fisher, Waltham, MA, USA).

1.2 Preparation of pure MOFs, biocomposites, and mixtures
1.2.1 Room-temperature synthesis of pure ZIF-67 and ZIF-8

Synthesis procedures were adapted from those reported by Gross *et al.* [1]. ZIF-67 structures with a 1:16:16 ratio of metal : ligand : TEA were prepared by first dissolving 0.717 g cobalt (II) nitrate (2.46 mmol) in 50 mL DI water. Then, a solution of 3.244 g HMe-Im (39.5 mmol) and 4.00 g TEA (39.52 mmol) in 50 mL DI water was stirred until dissolved. In the case of ZIF-8, the cobalt salt was substituted for 0.733 g zinc (II) nitrate (2.46 mmol). The cobalt or zinc solution was added to the HMe-Im/TEA solution, and the resulting mixture was stirred for 10 min. This mixture was then separated via centrifugation (3.0 RCF, 10 min), decanted, and suspended in DI water for 12 h. This centrifugation process was repeated for a second water wash, and after 12 h, the ZIF suspension was centrifuged again, and the solid was collected. Solid ZIF crystals were finally dried in vacuum for 2 h at 150℃ as described previously [1].

1.2.2 Room-temperature synthesis of BSA@ZIF-8 and BSA@ZIF-67

BSA@ZIF structures were prepared by first adding 40 mg of BSA to a solution of HMe-Im (160 mmol) in 20 mL DI water. A solution of cobalt (II) nitrate (40 mmol) or zinc (II) acetate (40 mmol) was then prepared in 20 mL DI water, and the solution was combined with the BSA solution and agitated to ensure thorough mixing as described in the work of Liang *et al.* [2]. The resulting mixture was aged overnight, separated *via* centrifugation (3.0 RCF, 10 min), and the supernatant was decanted. To remove residual BSA from the MOF crystals, the samples were washed four times: twice with water and twice with ethanol. Each wash cycle was completed by first adding 5 mL of wash solution to the MOF crystals and then agitating the solution until the crystals were fully suspended. The mixture was then sonicated for 10 min, centrifuged, and decanted before adding the next wash solution. After the final centrifugation, the sample was decanted and dried in ambient air (20 – 25˚C, 30 – 60% relative humidity) for 48 h.

Encapsulation efficiency (and therefore the BSA:MOF ratio) was quantified by taking samples post-centrifugation of the supernatant prior to decanting. These samples were analyzed using a BCA assay, which determined a consistent BSA encapsulation efficiency of ~80%.

1.2.3 Preparation of physical mixtures of BSA and MOF
Physical mixtures of lyophilized BSA and pure MOF components were prepared in BSA:MOF mass ratios of 1:9 (10% BSA) and 1:4 (20% BSA) by combining both powders and mixing thoroughly before suspending in 100 mM HEPES buffer in capillary tubes.

1.3 Confirming and quantifying degree of encapsulation
1.3.1 X-ray diffraction to confirm crystalline structure
Powder x-ray diffraction (pXRD) was used to confirm the structure of MOF crystals. For ZIF-8 samples, this was performed using a XPert Pro Alpha-1 diffractometer with X'Celerator detector using Cu $K\alpha_1$ radiation (λ=1.54184 Å) (Malvern Panalytical, Malvern, United Kingdom), as described previously [3].

For ZIF-67 samples, a PANalytical Empyrean diffractometer with PIXcel^{3D} detector using Cu $K\alpha_1$ radiation (λ=1.54184 Å) (Malvern) was used to reduce the effect background fluorescence and improve diffractogram visualization due to the presence of cobalt in the MOF. Biocomposites were ground into a powder with mortar and pestle before analysis. All scans were prepared on a zero-background holder and performed in room temperature ambient air.

1.3.2 FTIR to assess structural incorporation
Fourier-transform infrared spectroscopy (FTIR) measurements were done on a Nicolet iS10 FTIR spectrometer with the Smart iTX diamond attenuated total reflection (ATR) accessory (Thermo Fisher, Waltham, MA, USA). Pure MOFs, biocomposites, and lyophilized proteins were all analyzed as dry powders.

1.3.3 SEM for visualization of crystallites
Crystals were prepared by gold sputtering (Hummer 6 sputterer, Ladd Research, Williston, VT) for 5 minutes before visualization by scanning electron microscopy (SU8230, Hitachi, Tokyo, Japan). Samples were observed at magnifications ranging from 5,000x to 30,000x zoom at a voltage of 10.0 kV.

1.3.4 ELISA to validate surface wash protocol
A Bovine Serum Albumin ELISA kit (Alpha Diagnostic International, San Antonio, TX) was used to measure BSA concentration for intact and exfoliated biocomposites to assess the lack of surface-bound protein [4]. Biocomposite samples were exfoliated by adding 62 µL of 0.1 M EDTA to 1 mg of BSA@ZIF-8 suspended in 1 mL of dI H_2O. After the addition of EDTA, the samples were allowed to rest for 24 h and agitated frequently before dilution. All samples were diluted 6,000x in 1x phosphate-buffered saline before measurement with ELISA following the manufacturer's protocol.

2. Mathematical justification for subtraction approach

Although we adopted a trial-and-error approach for determining $\boldsymbol{\alpha}$ in this work, we also propose a mathematical basis for this scaling subtraction factor:

(1) $I_1 = \phi_1 I_p + \phi_2 I_m + [1 - (\phi_1 + \phi_2)] I_s$

(2) $I_2 = \phi_3 I_m + (1 - \phi_3) I_s$

(3) $I_1 - \boldsymbol{\alpha} I_2 = \phi_1 I_p + (1 - \phi_1 - \alpha I_s)$

Here, **Equation (1)** represents the scattered intensity from proteins within the MOF and the solvent. Here, I_p, I_m, and I_s are the scattered intensity contributions from proteins, MOF, and solvent, respectively, while ϕ_1 and ϕ_2 are the volume fractions of proteins and MOF in the scattering volume of the capillary tube. Similarly, the scattered intensity from the MOF suspended in solvent is determined by **Equation (2),** where ϕ_3 is the volume fraction of MOF in the scattering volume. By combining the first two equations and performing algebraic manipulation, we arrive at **Equation (3),** from which it is apparent that ϕ_1 is needed to obtain the actual scattering contribution from only the protein.

We do not have information about the volume fraction of the proteins in the scattering volume. Hence, without $\phi_1$we cannot separate the solvent background totally from the scattering contributions from proteins. While the proposed subtraction approach can remove the MOF scattering contribution, it cannot guarantee removal of the solvent scattering. In practice, we consider the solvent contribution to be relatively constant, and therefore treat it as a constant background on all analyses [5, 6]. Nonetheless, we predict that by knowing the volume fraction of each component in the scattering volume, the scaling subtraction factor $\boldsymbol{\alpha}$ could be determined mathematically with **Equation (3)**.

3. Supplementary Figures 1 to 5

Figure S1: ELISA measurement of intact and exfoliated BSA@ZIF-8

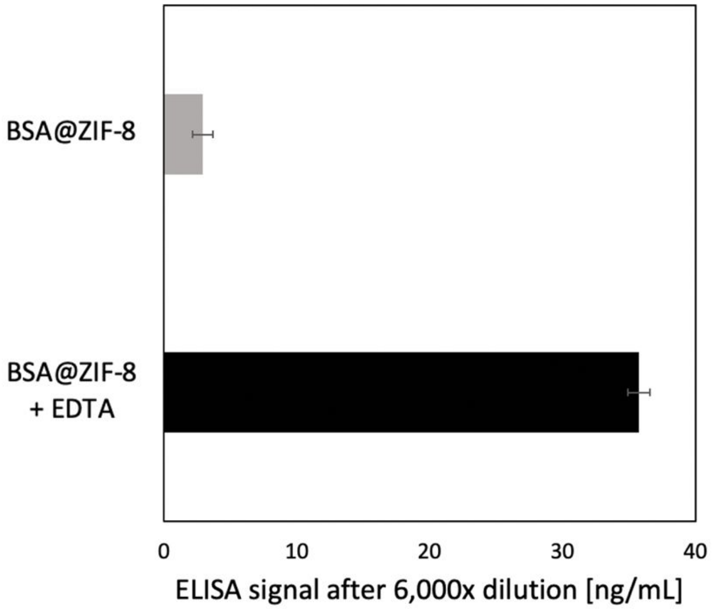

Figure S1: *Enzyme-linked immunosorbent assay of BSA concentrations for BSA@ZIF-8 before and after exfoliation by addition of 0.1M EDTA. Data represent the average ± standard deviation of 3 replicates.*

Figure S2: SEM for pure ZIF-67 and ZIF-8 crystals

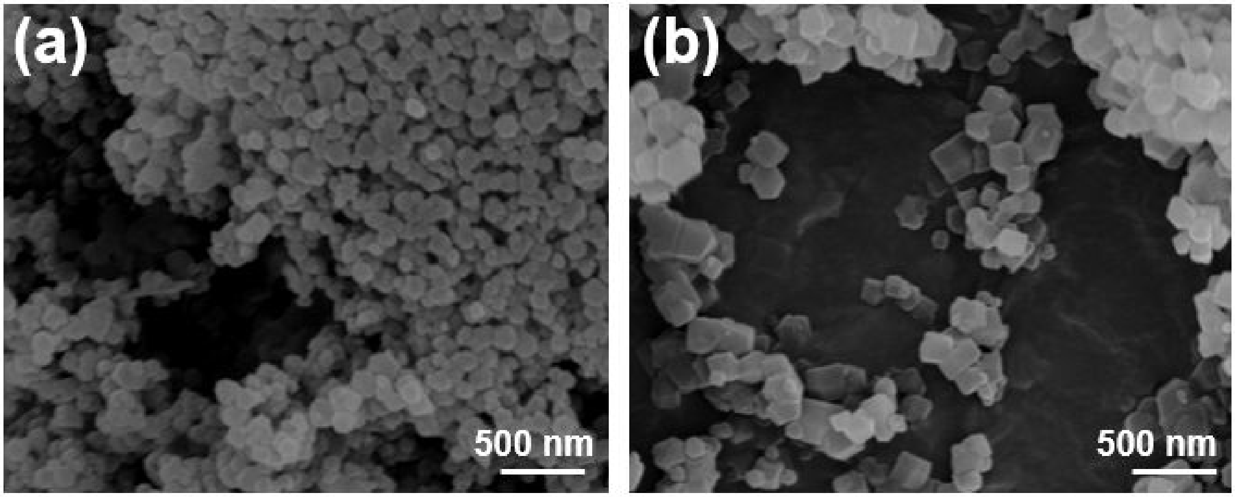

Figure S2*: Representative SEM images of (a) pure ZIF-67 crystallites and (b) pure ZIF-8 crystallites. Both images were taken at 30,000x magnification and scale bars represents 500 nm.*

Figure S3: PDDFs for native and MOF-encapsulated BSA

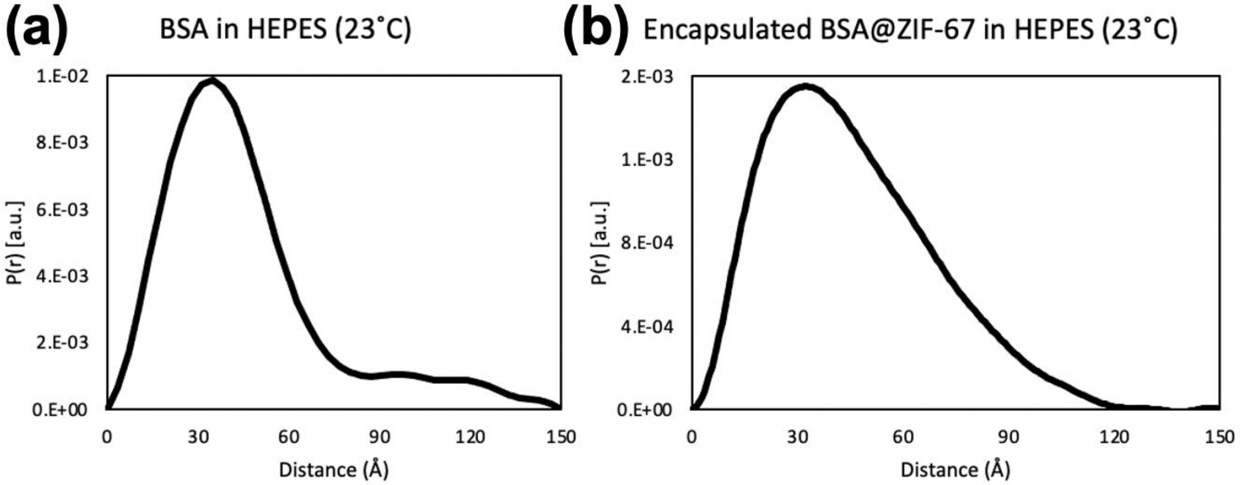

***Figure S3**: Representative PDDFs for (a) native BSA and (b) BSA encapsulated in ZIF-67 with a D_{max} value set to 150 Å. Native BSA shows a broad, low-intensity peak around 100 Å, indicating the presence of BSA oligomers. Conversely, encapsulated BSA has a single broad peak with no secondary peaks, suggesting the exclusive presence of monomeric BSA (i.e., suggesting just one BSA molecule per MOF cavity).*

Figure S4: Guinier fits for BSA@ZIF-67 and native BSA

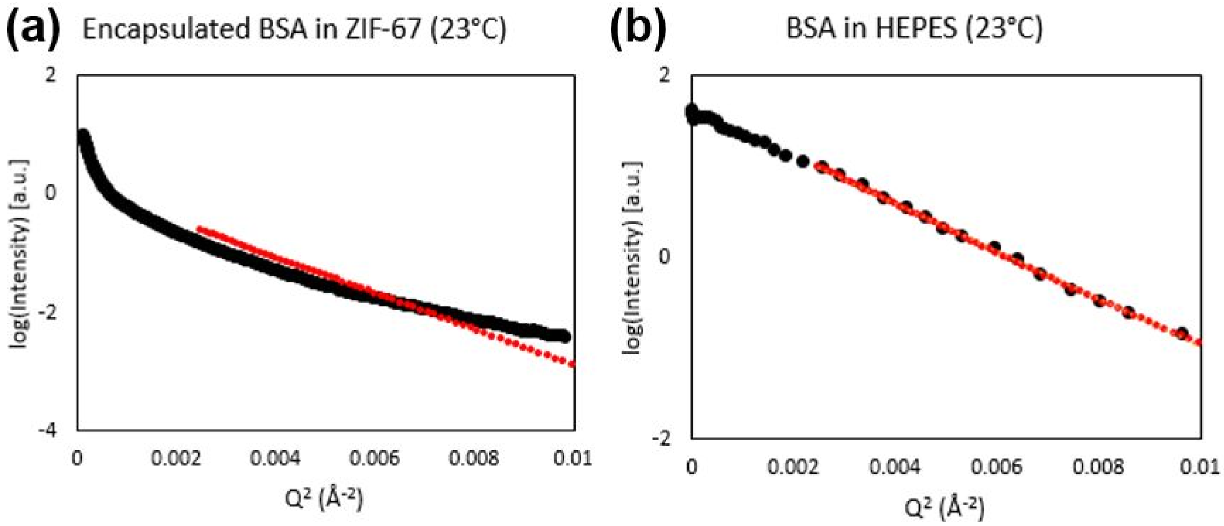

Figure S4: *Representative Guinier approximations (red) for raw SAXS spectra (black) of (a) BSA encapsulated in ZIF-67 and (b) native BSA in HEPES buffer taken at room temperature. Calculated R_g values were 30.25 Å for encapsulated BSA and 28.05 Å for BSA in HEPES.*

Figure S5: PDDF for 10% BSA + ZIF-67 mixture

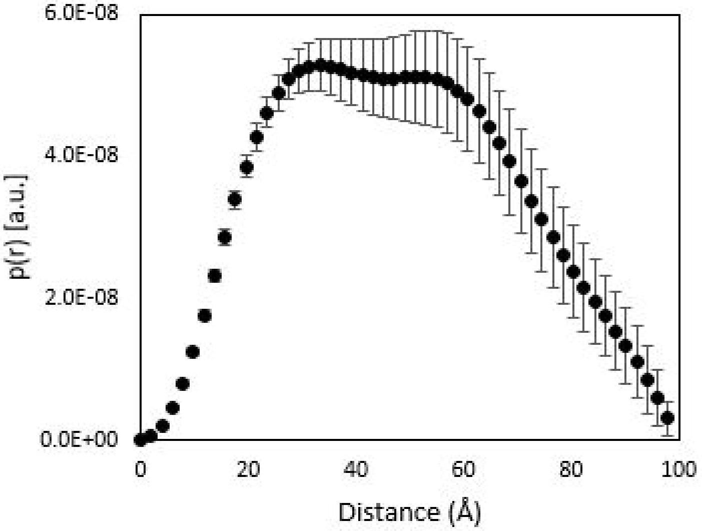

Figure S5: *Representative PDDF from calculated spectra of a physical mixture of BSA and ZIF-67 prepared at a BSA:MOF ratio of 1:9 (10% BSA). Predicted R_g value from PDDF was 37.3 Å This result is inconsistent with successful subtraction as the expected globular shape is not observed.*

Works Cited

[1] Gross, A.F., E. Sherman, and J.J. Vajo, Aqueous room temperature synthesis of cobalt and zinc sodalite zeolitic imidizolate frameworks. Dalton Transactions, 2012. 41(18): p. 5458-5460.
[2] Liang, K., et al., Biomimetic mineralization of metal-organic frameworks as protective coatings for biomacromolecules. Nature Communications, 2015. 6(1): p. 7240.
[3] Shade, D., B. Marszalek, and K.S. Walton, Structural similarity, synthesis, and adsorption properties of aluminum-based metal-organic frameworks. Adsorption, 2021. 27(2): p. 227-236.
[4] Bovine Albumin (BSA) ELISA kit, 96 tests, Quantitative. Alpha Diagnostics International, San Antonio, TX, 2023.
[5] Fan, L.X., et al., The Absolute Calibration of a Small-Angle Scattering Instrument with a Laboratory X-ray Source. Xiv International Conference on Small-Angle Scattering (Sas09), 2010. 247.
[6] Clark, G.N.I., et al., Small-angle scattering and the structure of ambient liquid water. Proceedings of the National Academy of Sciences of the United States of America, 2010. 107(32): p. 14003-14007.
